# The NLRP3 inflammasome inhibitor OLT1177 rescues cognitive impairment in a mouse model of Alzheimer’s disease

**DOI:** 10.1073/pnas.2009680117

**Published:** 2020-11-30

**Authors:** Niklas Lonnemann, Shirin Hosseini, Carlo Marchetti, Damaris B. Skouras, Davide Stefanoni, Angelo D’Alessandro, Charles A. Dinarello, Martin Korte

**Affiliations:** ^a^Department of Cellular Neurobiology, Zoological Institute, Technische Universität Braunschweig, 38106 Braunschweig, Germany;; ^b^Neuroinflammation and Neurodegeneration Group, Helmholtz Centre for Infection Research, 38124 Braunschweig, Germany;; ^c^Department of Medicine, University of Colorado, Denver, Aurora, CO 80045;; ^d^R&D Division, Olatec Therapeutics, New York, NY 10065;; ^e^Department of Biochemistry and Molecular Genetics, University of Colorado, Denver, Aurora, CO 80045;; ^f^Department of Medicine, Radboud University, Medical Center, 6525 Nijmegen, The Netherlands

**Keywords:** synaptic plasticity, Alzheimer’s disease synaptic, cognitive function

## Abstract

Numerous studies demonstrate that neuroinflammation is a key player in the progression of Alzheimer’s disease (AD). Interleukin (IL)-1β is a main inducer of inflammation and therefore a prime target for therapeutic options. The inactive IL-1β precursor requires processing by the the nucleotide-binding oligomerization domain-like receptor family, pyrin domain containing 3 (NLRP3) inflammasome into a mature and active form. Studies have shown that IL-1β is up-regulated in brains of patients with AD, and that genetic inactivation of the NLRP3 inflammasome improves behavioral tests and synaptic plasticity phenotypes in a murine model of the disease. In the present study, we analyzed the effect of pharmacological inhibition of the NLRP3 inflammasome using dapansutrile (OLT1177), an oral NLRP3-specific inhibitor that is safe in humans. Six-month-old WT and APP/PS1 mice were fed with standard mouse chow or OLT1177-enriched chow for 3 mo. The Morris water maze test revealed an impaired learning and memory ability of 9-mo-old APP/PS1 mice (*P* = 0.001), which was completely rescued by OLT1177 fed to mice (*P* = 0.008 to untreated APP/PS1). Furthermore, our findings revealed that 3 mo of OLT1177 diet can rescue synaptic plasticity in this mouse model of AD (*P* = 0.007 to untreated APP/PS1). In addition, microglia were less activated (*P* = 0.07) and the number of plaques was reduced in the cortex (*P* = 0.03) following NLRP3 inhibition with OLT1177 administration. We also observed an OLT1177 dose-dependent normalization of plasma metabolic markers of AD to those of WT mice. This study suggests the therapeutic potential of treating neuroinflammation with an oral inhibitor of the NLRP3 inflammasome.

## Significance

IL-1β is an immunomodulatory cytokine that is overexpressed in the brains of patients with Alzheimer’s disease (AD). The NLRP3 inflammasome is an intracellular complex that activates caspase-1, which processes the IL-1β and IL-18 precursors into active molecules. In this study, we used an APP/PS1 mouse model for AD, which confirms significant cognitive losses that are recovered in NLRP3-deficient mice, to evaluate the therapeutic potential of an orally bioavailable and safe NLRP3 inhibitor, OLT1177. OLT1177 ameliorated the phenotype in APP/PS1 mice, as evidenced by rescued spatial learning and memory in the Morris water maze test. Microglia were less activated, cortical plaques reduced, and plasma AD metabolic markers were normalized. OLT1177 is a potential therapeutic option for AD.

Alzheimer’s disease (AD) and other related neurodegenerative diseases leading to dementia represent an enormous burden for the society and health economies. AD patients suffer progressive cognitive and functional deficits often for many years, which result in a heavy burden to patients, families, and the public health system. In fact, in 2015 an estimated 46.8 million people worldwide were living with dementia, which could extend to 131.5 million by 2050 (1). Rising prevalence and mortality rates in combination with a lack of effective treatments lead to enormous costs to society. Research on AD in the last decades has focused on the pathological hallmarks and cellular deposits of amyloid-β (Aβ) peptides and neurofibrils (2). Recently, there has been increased evidence supporting a central role of the immune system in the progression or even the origin of the disease (3–5). In this respect, it is noteworthy that it has been known since 1989 that levels of interleukin (IL)-1β, one of the main mediators of innate immune response, are elevated in brains of patients with AD and can be associated with the progression and onset of AD (6–11). Additionally, it was shown that the nucleotide-binding oligomerization domain-like receptor family, pyrin domain containing 3 (NLRP3) inflammasome (12, 13), a multisubunit complex important for the maturation of IL-1β, is activated by Aβ peptides, leading to an overproduction of IL-1β, neuroinflammation, and cognitive impairment (14, 15). Inhibition of the NLRP3 inflammasome and the subsequent reduced IL-1β production can be linked to a change in the phenotype of microglia, the innate immune cells in the brain. Heneka et al. (16) pointed out the important role of the NLRP3 inflammasome/caspase-1 axis in AD pathogenesis by demonstrating significant improvements (e.g., in cognition) in APP/PS1 mice (a mouse model for AD) when crossed with NLRP3^−/−^ animals. The APP/PS1 mice express a human amyloid precursor protein (APP) and human presenilin-1 (PS1), leading to the accumulation of Aβ peptides, neuroinflammation, and cognitive impairment (17).

OLT1177 (rINN: dapansutrile) is a new chemical entity small molecule that specifically targets the NLRP3 inflammasome and prevents the activation of caspase-1 and the maturation and release of IL-1β (18). OLT1177 has been shown to be well tolerated in animals and humans (18) and is currently in phase 2 clinical studies for the treatment of inflammatory conditions, such as osteoarthritis (topical gel dosage form) and inflammatory diseases, such as acute gout flare (oral capsule dosage form), among other diseases (19).

In this study, we used the APP/PS1 mouse model of AD to investigate the effects of OLT1177 as an acute, oral pharmacological intervention (17). Six-month-old WT and APP/PS1ΔE9 mice consumed ad libitum OLT1177 in feed pellets (∼0, 500, or 1,000 mg/kg/d based on feed concentrations of 0, 3.75 or 7.5 g of OLT1177 per kilogram of feed; hereafter referred to as 3.75 or 7.5 g/kg OLT1177) for the treatment duration of 3 mo. APP/PS1 mice treated with OLT1177 showed rescue effects in various assessments, ranging from improved cognitive function to overall reduction in proinflammatory cytokines in the brain, suggesting the potential benefits of pharmaceutically blocking NLRP3 signaling in AD.

## Results

### Recovered Cognitive Phenotype in the Spatial Memory Test in 9-mo-old APP/PS1 Mice Treated with OLT1177.

**Fig. 1.**
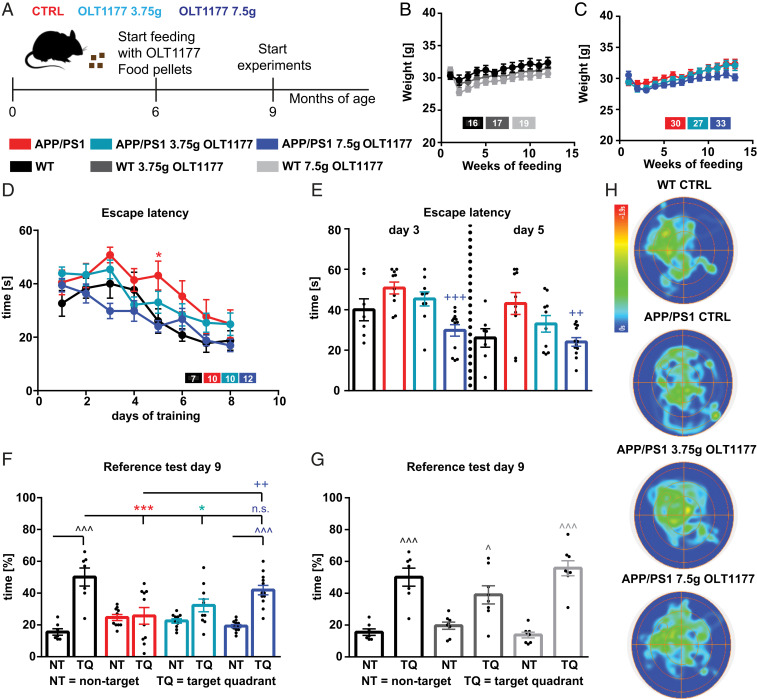
Oral administration of OLT1177 in APP/PS1 animals for 3 mo restores cognitive deficits. (*A*) The treatment of either OLT1177 (3.75 g/kg and 7.5 g/kg; drug per kilogram feed) or control food was started at the age of 6 mo and continued for 3 mo. (*B* and *C*) During the treatment period, neither WT nor APP/PS1 mice show differences in weight (*n* = 16 to 33 animals). (*D*) WT and APP/PS1 mice indicate a learning behavior during the training phase of the spatial learning test. APP/PS1 mice show higher escape latency during acquisition on day 5 compared to WT mice (day 5 *P* = 0.037; *n* = 7 to 12 animals). (*E*) Moreover, on days 3 and 5 the APP/PS1 mice show increased escape latency compared to APP/PS1 mice treated with 7.5 g/kg OLT1177 (day 3 *P* = 0.001; day 5 *P* = 0.007). (*F*) WT mice and APP/PS1 mice treated with 7.5 g/kg OLT1177 display a significant preference for the TQ, whereas the APP/PS1 mice with control or low dose food did not show any preference (NT vs. TQ: WT *P* < 0.001; APP/PS1 *P* > 0.99; APP/PS1 3.75 g/kg *P* = 0.39; APP/PS1 7.5 g/kg *P* < 0.001; WT vs. APP/PS1 *P* = 0.001; WT vs. APP/PS1 3.75 g/kg *P* = 0.014; WT vs. APP/PS1 7.5 g/kg *P* = 0.68; APP/PS1 vs. APP/PS1 7.5 g/kg *P* = 0.0088). (*G*) WT animals treated with control food, low-dose, or high-dose OLT1177 did not show any differences between the groups. (*H*) The heat maps of pooled animals manifest the results of the reference test (scale: blue 0 s to red 1.9 s, *n* = 3 to 4 animals). Data are presented as mean ± SEM. *P < 0.05, ***P < 0.001 compared to WT, *^++^P *< 0.01*,* ^+++^*P* < 0.001 compared to APP/PS1 CTRL, ^*P *< 0.05, ^^^*P* < 0.001 compared to NT.

We first evaluated whether OLT1177-enriched diets had any obvious effect on the phenotype of mice. Following 3 mo of either a standard or OLT1177 diet (3.75 g/kg and 7.5 g/kg) (Fig. 1*A*), WT and APP/PS1 mice showed no significant change in weight (Fig. 1 *B* and *C*).

The Morris water maze (MWM) test (20) was performed to investigate whether the cognitive deficits of APP/PS1 mice were rescued when treated orally with the NLRP3 inhibitor OLT1177. The MWM test revealed that the escape latency was progressively decreased in all tested groups during the 8-d acquisition period (Fig. 1*D*). However, APP/PS1 animals treated with control food showed an elevated escape latency during the 8-d acquisition compared to WT mice (Fig. 1*D*). Especially on day 5, the APP/PS1 animals showed a significantly higher escape latency compared to WT mice (Fig. 1*D*). In contrast, APP/PS1 mice treated with 7.5 g/kg OLT1177 demonstrated significantly diminished escape latency on days 3 and 5 compared to untreated APP/PS1 mice (Fig. 1*E*). It is noteworthy that the escape latency in APP/PS1 mice treated with 7.5 g/kg OLT1177 did not show any significant differences compared to WT animals. APP/PS1 mice on the 3.75 g/kg OLT1177 diet did not demonstrate a decrease in the escape latency relative to untreated APP/PS1 mice.

Furthermore, to provide evidence for spatial learning, on day 3 and day 9 of acquisition prior to the training and 24 h after the last training session, a reference memory test was performed (probe trial) where the mice were tested without a platform to escape. Here, we compared the mean time in percentage of the three nontarget quadrants (NT) to the percent time spent in the target quadrant (TQ, where the platform was located during training period). On day 9, the results showed a significant preference for the TQ in WT and APP/PS1 mice treated with 7.5 g/kg OLT1177, whereas no TQ preference was detected in untreated APP/PS1 mice and APP/PS1 treated with 3.75 g/kg OLT1177 (Fig. 1*F*). The results of MWM test did not reveal any significant differences between WT mice receiving control and both doses (3.75 and 7.5 g/kg) of OLT1177 (Fig. 1*G*); thus, it can be concluded that OLT1177 treatment does not have any negative side effects on WT mice. In order to clarify the performance in the probe trial, the pooled heat maps of the groups were analyzed. These results also show a longer time in the TQ for WT and APP/PS1 7.5g/kg animals (Fig. 1*H*). Taken together, these data confirm an impaired learning and memory ability in APP/PS1 mice, which was previously reported by Heneka et al. (16), a phenotype that can be completely rescued following 3-mo oral administration with 7.5 g/kg OLT1177.

### Recovered Phenotype in Electrophysiological Experiments in 9-mo-old APP/PS1 Mice Treated with OLT1177.

Given the observed recovery in the impaired learning and memory ability of APP/PS1 mice treated with 7.5 g/kg OLT1177, we determined whether hippocampal network function would also be improved (altered) following 3 mo of OLT1177 administration. For this purpose, we analyzed synaptic plasticity at the Schaffer collateral pathway connecting the CA3 with the CA1 subregions, one of the most extensively studied synapses in the CNS, as reviewed in Korte and Schmitz (21). In addition, to investigate the potential therapeutic effect of OLT1177 on hippocampal function in APP/PS1 mice and to ensure that the administration of either 3.75 g/kg or 7.5 g/kg OLT1177 does not have negative side effects on hippocampal function, basal synaptic transmission and short- and long-term synaptic plasticity separately were compared between control mice treated with control food and mice treated with low and high doses of OLT1177.

**Fig. 2.**
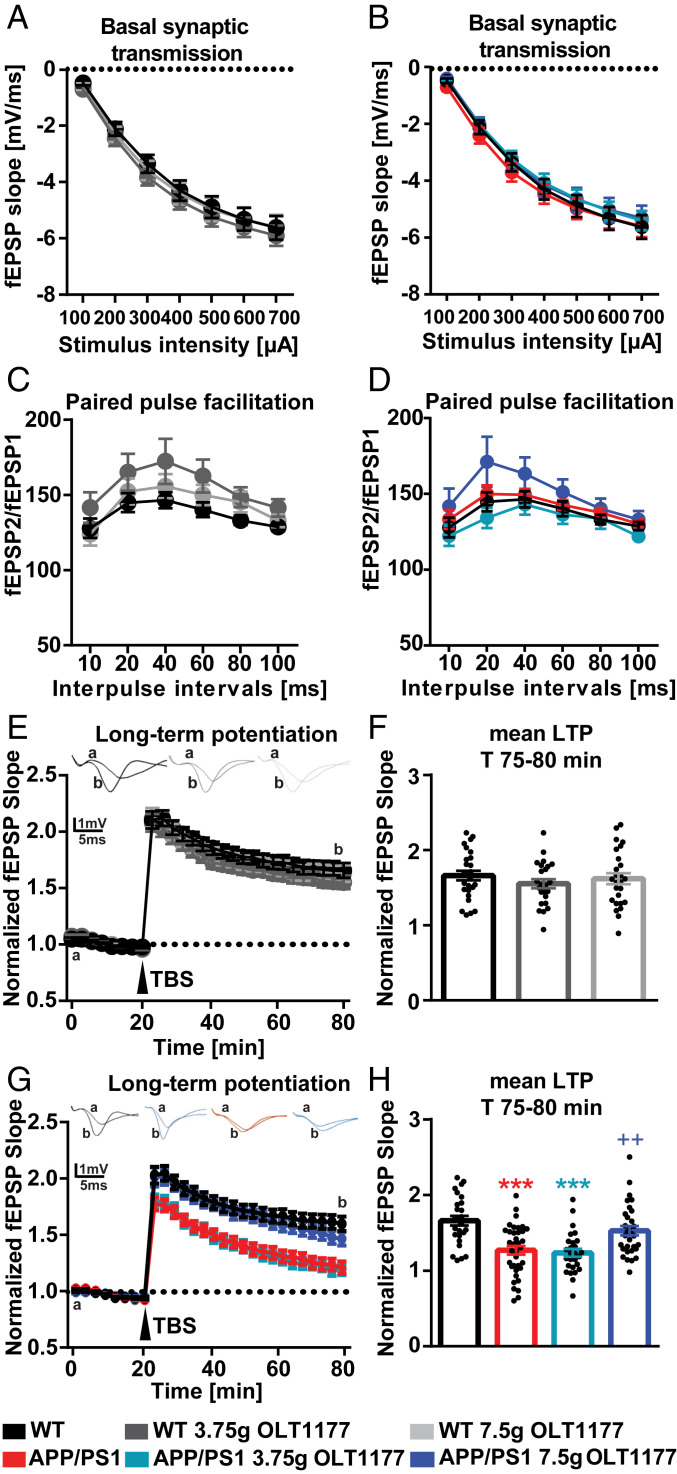
Oral administration of OLT1177 in APP/PS1 animals for 3 mo rescues synaptic plasticity impairment to WT conditions. (*A* and *B*) WT and APP/PS1 mice treated either with control food or OLT1177 did not show any differences in the properties of basal synaptic transmission (*A*, *P* = 0.72; *B*, *P* = 0.843) and (*C* and *D*) ratios of pared-pulse facilitation (*C*, *P* = 0.22; *D*, *P* = 0.19). (*E*) WT mice treated either with control food or OLT1177 exhibited the same magnitude of LTP in response to strong afferent stimulation (LTP was induced by θ-burst stimulation: TBS, four bursts at 100 Hz repeated 10 times in a 200-ms interval, repeated three times in a 10-s interval; denoted with an arrow) (*P* = 0.51). (*F*) Comparison of mean values (average of last 5 min of recordings) LTP magnitude in control mice summarized as bar graphs (*P* = 0.52). (*G*) APP/PS1 mice treated with either control food or low dose of OLT1177 showed a significant impairment in LTP induced by TBS compared to control group; however, administration of OLT1177 at high dose could rescue the phenotype in APP/PS1 mice (*P* < 0.001). (*H*) The mean LTP magnitude (average of 55 to 60 min after TBS) was significantly lower in APP/PS1 mice treated either with control food or 3.75 mg/kg of OLT1177. Administration of OLT1177 at high dose could rescue the phenotype in APP/PS1 mice (CTRL: 1.661 ± 0.03; APP/PS1: 1.27 ± 0.05; APP/PS1-low dose: 1.23 ± 0.05; and APP/PS1-high dose: 1.52 ± 0.06, *P* < 0.001). In (*E*) and (*G*) *a* represents the baseline curve and *b* represents the curve after TBS induction. Data are presented as mean ± SEM. ***P < 0.001 compared to WT, ^++^*P* < 0.01 compared to APP/PS1 (*n* = 6 to 7 animals and *n* = number of slices in each group = 24 to 36).

To assess the basal synaptic transmission, input–output strength at a defined stimulus intensity in WT and APP/PS1 mice and APP/PS1 mice treated with 3.75 and 7.5 g/kg OLT1177 was measured (Fig. 2 *A* and *B*). No significant difference of field excitatory postsynaptic potential (fEPSP) slope size was detected between each of the tested groups (Fig. 2*B*). In addition, the investigation of the presynaptic function by paired-pulse facilitation showed no differences between groups (Fig. 2*D*).

The slices taken from WT mice treated with 3.75 and 7.5 g/kg OLT1177 had no detectable deficit in long-term potentiation (LTP) compared to mice treated with control food (Fig. 2*E*). The mean value of the maintenance phase of LTP (T 75 to 80 min) was comparable between WT mice treated with control food (1.66 ± 0.06), 3.75 g/kg (1.55 ± 0.06), and 7.5 g/kg (1.61 ± 0.37) OLT1177 (Fig. 2*F*). Taken together, these findings indicated that administration of either dose of OLT1177 does not show any negative side effects on hippocampal function, as was the case for the assessment of learning and memory.

To study the possible therapeutic effects of OLT1177 in processes of long-term synaptic plasticity, LTP was analyzed in treated APP/PS1 and control mice. Slices taken from APP/PS1 mice fed either with control food or feed containing 3.75 g/kg OLT1177 showed a significantly impaired LTP compared to the WT control animals. However, the impairment of LTP in APP/PS1 mice was rescued following administration of 7.5 g/kg OLT1177 (Fig. 2*G*). Similarly, the comparison of the mean value of the maintenance phase of LTP (displayed as the last 5 min of the recording) between different experimental groups supports this conclusion (Fig. 2*H*). Taken together, these results indicate that a dose of 3.75 g/kg OLT1177 was not able to improve LTP deficits of APP/PS1 mice. However, a dose of 7.5 g/kg OLT1177 was sufficient to rescue the defect.

### Recovered Phenotype in Neuronal Morphology Assessments in 9-mo-old APP/PS1 Mice Treated with OLT1177.

**Fig. 3.**
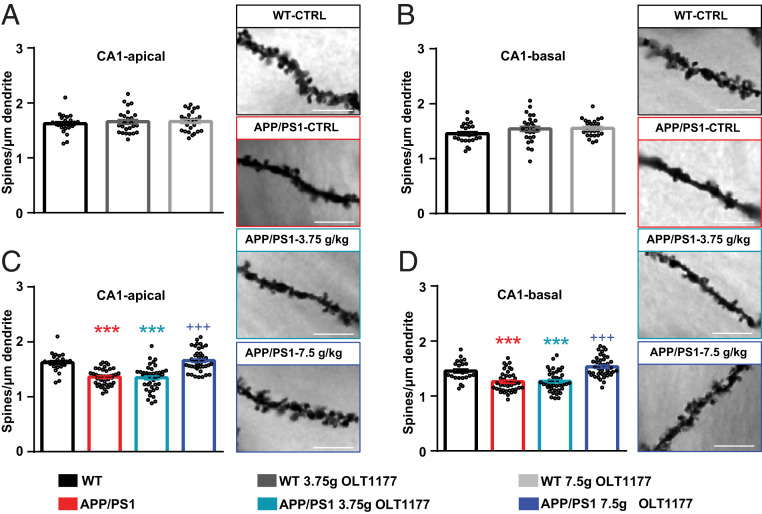
Oral administration of OLT1177 in APP/PS1 animals for 3 mo restores dendritic spine loss to WT conditions. (*A* and *B*) Dendritic spine density of CA1-apical and -basal was not changed in WT mice following administration of 3.75 g/kg and 7.5 g/kg OLT1177. (*C* and *D*) Spine density in both apical and basal dendrites of CA1 hippocampal neuron was significantly diminished in APP/PS1 mice treated either with control food or 3.75 g/kg OLT1177. Administration of 7.5 g/kg OLT1177 could rescue the phenotype in APP/PS1 mice (WT vs. APP/PS1 *P* = 0.001, WT vs. APP/PS1 3.75 g/kg *P* = 0.001, WT vs. APP/PS1 7.5 g/kg *P* = 0.89, APP/PS1 vs. APP/PS1 7.5 g/kg *P* = 0.001 in *C*; WT vs. APP/PS1 *P* = 0.001, WT vs. APP/PS1 3.75 g/kg *P* = 0.001, WT vs. APP/PS1 7.5 g/kg *P* = 0.36, APP/PS1 vs. APP/PS1 7.5 g/kg *P* = 0.001 in *D*). Representative images of dendritic spines of hippocampal CA1 neurons in the tested groups are presented. (Scale bars, 5 µm.) Data are presented as mean ± SEM. ***P < 0.001 compared to WT, ^+++^*P* < 0.001 compared to APP/PS1 (*n* = 5 animals and *n* = 8 dendrites).

To investigate the observed rescue of impaired spatial learning and defects in LTP in APP/PS1 mice administered OLT1177 at a cellular level, hippocampal neuron morphology was blindly analyzed in each experimental group (Fig. 3). For this analysis we focused on spines, tiny dendritic protrusions that receive postsynaptic excitatory input in the hippocampus and neocortex. In addition, alterations in dendritic spine density and morphology have been shown to correlate with defects in synaptic plasticity and cognitive function (22). Therefore, dendritic spines were counted on apical and basal dendrites of CA1 pyramidal neurons in the hippocampus (*SI Appendix*, Fig. S1). To ensure that the administration of either 3.75 g/kg or 7.5 g/kg of OLT1177 does not have any side effects on the morphology of hippocampal neurons of WT animals, dendritic spine density was compared between WT mice treated with control food or feed enriched with OLT1177. Since the spine density in CA1-apical (Fig. 3*A*) and basal dendrites (Fig. 3*B*) did not reveal any significant differences between the tested groups, we concluded that the administration of OLT1177 up to 7.5 g/kg in feed does not negatively affect the structure of hippocampal neurons.

Further assessment of dendritic spine density in APP/PS1 mice following administration of OLT1177 was performed. A significant reduction in spine density of apical (Fig. 3*C*) and basal (Fig. 3*D*) dendrites of CA1 pyramidal neurons was observed in APP/PS1 mice treated either with control food or a dose of 3.75 g/kg OLT1177 compared to WT; however, this reduction was rescued in APP/PS1 mice following administration of a higher dose of OLT1177 (7.5 g/kg). Taken together, these results revealed that the administration of 7.5 g/kg OLT1177 was able to rescue the decreased dendritic spine phenotype in APP/PS1 mice; however, this was not the case for the lower dose of OLT1177 (3.75 g/kg).

### Treatment with OLT1177 Reduced Microglia Activation and the Number of Plaques in the Cortex.

**Fig. 4.**
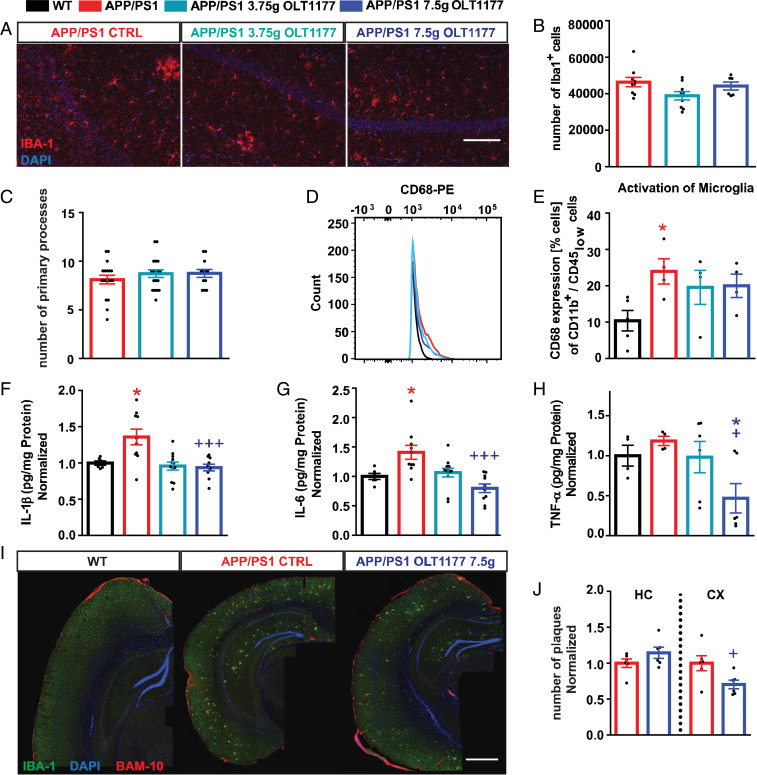
The administration of oral OLT1177 in APP/PS1 animals for 3 mo significantly reduces microglia activation. (*A*–*C*) Evaluation of microglial cells by immunostaining of IBA-1 (IBA-1 in red, DAPI in blue) showed no significant differences in activation status of microglial cells between APP/PS1 animals treated with OLT1177 or without (*n* = 6 to 9, *B*; *n* = 12 to 18, *C*). (Scale bar in *A,* 100 µm.) (*D* and *E*) In addition the percentage of CD68 expressing cells, as an activation marker for microglial cells, was enhanced in CD11b^+^/CD45^low^ gated cells of APP/PS1 mice treated with control food (WT vs. APP/PS1 *P* = 0.015, *n* = 4 to 5 animals). (*F*–*H*) Elevation of proinflammatory cytokines IL-1β, IL-6, and TNF-α was detected in brain homogenates of APP/PS1 mice (WT vs. APP/PS1 *P* = 0.019, APP/PS1 vs. APP/PS1 7.5 g/kg *P* = 0.001, *F*; WT vs. APP/PS1 *P* = 0.039, APP/PS1 vs. APP/PS1 7.5 g/kg *P* = 0.01, *G*; APP/PS1 vs. APP/PS1 7.5 g/kg OLT1177 *P* = 0.011, *H*; *n* = 6 to 12 samples). (*I*) The amount of Aβ plaques was determined using immunostaining for IBA-1 and BAM-10 (clone for Aβ) (IBA-1 in green, DAPI in blue, BAM-10 in red). (Scale bar in *I*, 500 µm.) (*J*) Plaque load was lower in the cortex of the APP/PS1 animals fed with 7.5 g/kg OLT1177 compared to APP/PS1 animals (cortex [CX] *P* = 0.03; hippocampus [HC] *P* = 0.16, *n* = 6 in both groups). Data are presented as mean ± SEM. *P < 0.05 compared to WT, ^+^*P *< 0.05, ^+++^*P* < 0.001 compared to APP/PS1 CTRL.

To estimate whether the rescued learning ability by OLT1177 and underlying cellular mechanisms were associated with a change in inflammation, the activation status of microglia was examined. First, the total number of microglial cells and their primary processes, as an activation hallmark, were analyzed using IBA-1 staining (Fig. 4*A*). Our findings did not reveal any significant differences in the number of microglia (Fig. 4*B*) and their amount of primary processes (Fig. 4*C*) in the CA1 area of the hippocampus in all tested groups. To assess neuroinflammation in more detail, the whole brains of untreated and OLT1177-treated mice were homogenized and examined either for the presence of proinflammatory cytokines or, using single-cell suspensions, for microglia activation via CD68 expression. An elevated activation of microglial cells in APP/PS1 mice compared to WT animals was found. In contrast, no significant enhanced CD68 expression was observed for APP/PS1 mice treated with 3.75 g/kg and 7.5 g/kg OLT1177 (Fig. 4 *D* and *E*). The analysis of proinflammatory cytokines showed significantly increased levels of IL-1β (Fig. 4*F*) and IL-6 (Fig. 4*G*) in APP/PS1 mice treated with control food, whereas no elevated levels of these cytokines were found in untreated WT mice and in APP/PS1 mice treated with 7.5 g/kg OLT1177. Even TNF-α levels (Fig. 4*H*) showed significantly reduced levels in untreated APP/PS1 mice compared with APP/PS1 mice treated with 7.5 g/kg OLT1177. In summary, an increase in all tested proinflammatory cytokines was found in untreated APP/PS1 mice, which was abolished by the oral administration of 7.5 g/kg OLT1177 (Fig. 4 *F*–*H*).

Finally, to investigate whether the reduction in neuroinflammation observed in the brains of APP/PS1 mice following NLRP3 inhibition with OLT1177 influenced the amount of Aβ plaques in the parenchyma of the CNS, the cortex and the hippocampus were evaluated by immunohistochemistry (Fig. 4*I*). While no differences were detected in the hippocampus, a reduced Aβ plaque load was found in the cortex of APP/PS1 animals administered 7.5 g/kg OLT1177 (Fig. 4*J*).

### Reduced Inflammatory Response in Primary Microglia Cells Treated with OLT1177 after LPS Stimulation.

**Fig. 5.**
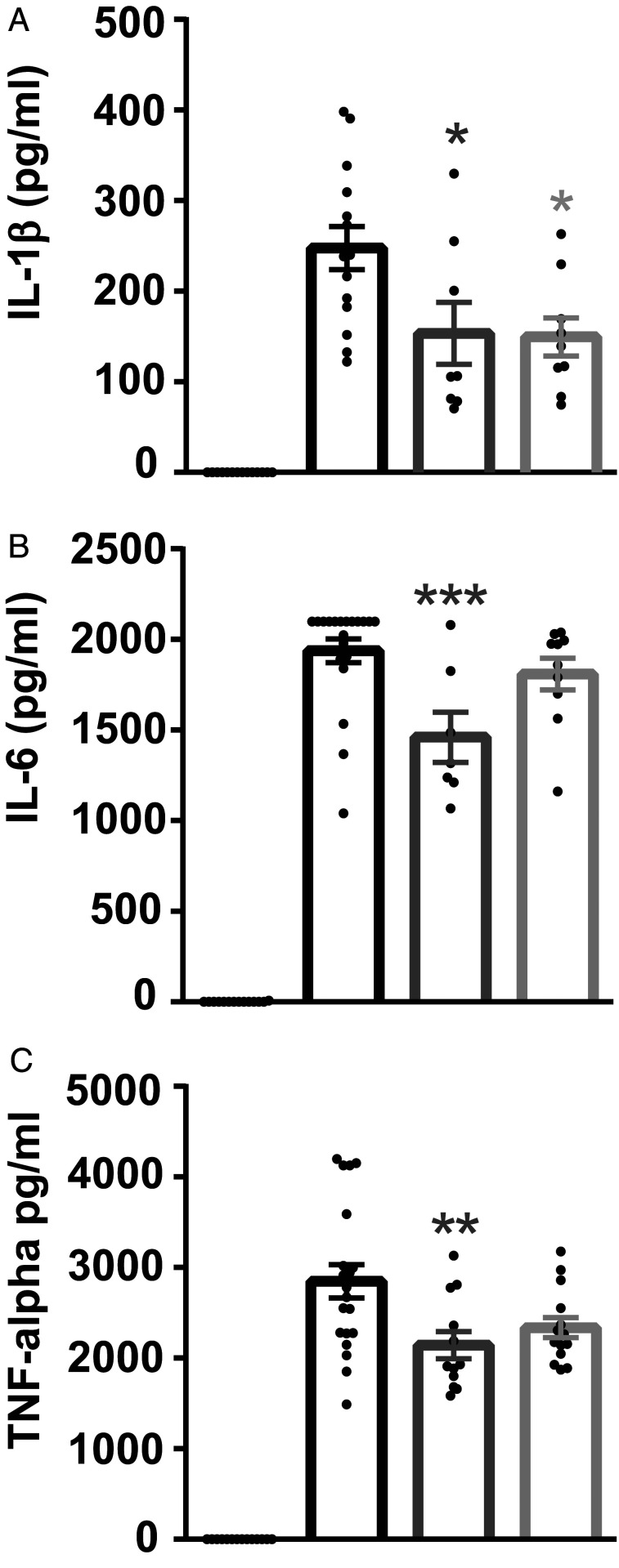
Direct effects of OLT1177 on microglia cells in vitro. WT primary microglia cells were cultured and stimulated with 1 µg/mL LPS (*E. coli*) with or without OLT1177 (5 µM or 10 µM) for 24 h. IL-1β (*A*), IL-6 (*B*), and TNF-α (*C*) were measured in the supernatant. Microglia cells treated with 5 µM OLT1177 showed a significantly reduced release of all three cytokines compared to cells treated with LPS alone (IL-1β LPS vs. LPS 5 µM OLT *P* = 0.02; LPS vs. LPS 10 µM OLT *P* = 0.01, *A*; IL-6 LPS vs. LPS 5 µM OLT *P* = 0.001, LPS vs. LPS 10 µM OLT *P* = 0.57, *B*; TNF-α LPS vs. LPS 5 µM OLT *P* = 0.006; LPS vs. LPS 10 µM OLT *P* = 0.054, *C*). Data are presented as mean ± SEM. *P < 0.05, **P < 0.01, ***P < 0.001 compared to WT microglia stimulated with LPS.

Next, in vitro studies were performed to assess the effect of OLT1177 administration on microglia in an inflammatory state (Fig. 5). WT primary microglia cells were cultured and stimulated with 1 µg/mL LPS (*Escherichia coli*) with or without OLT1177 (5 µM or 10 µM) for 24 h. The proinflammatory cytokines IL-1β, IL-6, and TNF-α were measured in the supernatant. Microglia cells treated with 5 µM OLT1177 showed a significantly reduced release of each of these cytokines compared to cells treated with LPS alone (Fig. 5), supporting the hypothesis that OLT1177 leads to a rescue of the AD phenotype in APP/PS1 mice via a reduction of a general proinflammatory response at the cellular level in the CNS.

### APP/PS1 Mice Show Increases in Plasma Metabolic Markers of AD That Are Normalized by OLT1177 Treatment.

**Fig. 6.**
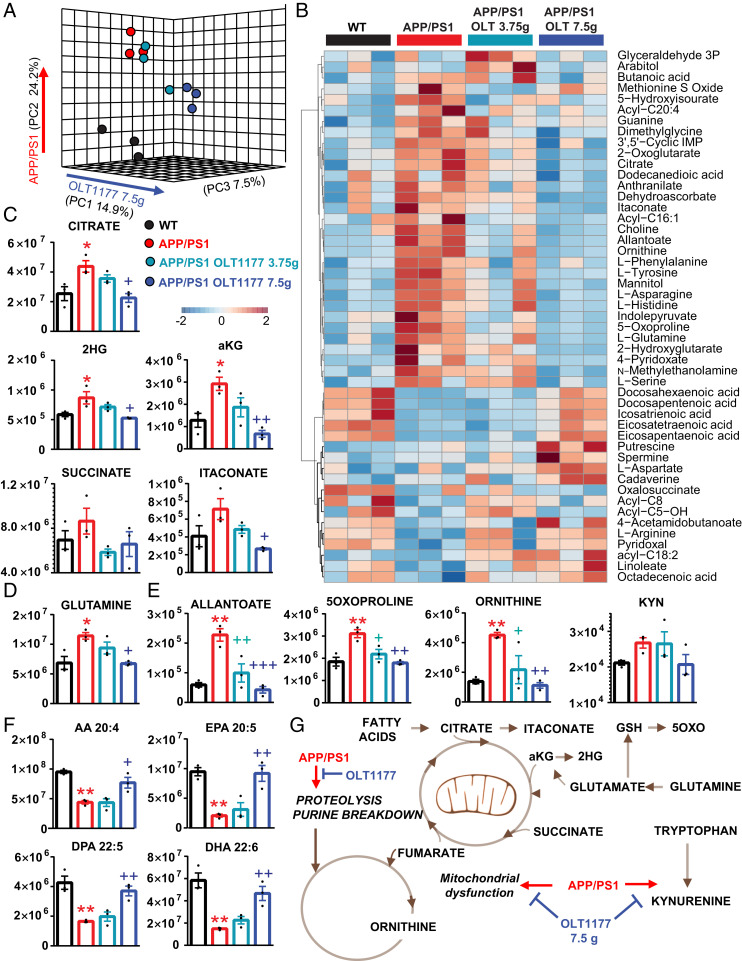
APP/PS1 mice show increases in plasma metabolic markers of AD that are normalized by OLT1177 treatment. Metabolomics analyses were performed on plasma from WT and APP/PS1 mice, either untreated or fed 3.75 or 7.5 g/kg OLT1177. (*A*) Multivariate analyses of metabolomics data, including partial least-square discriminant analysis and (*B*) hierarchical clustering analysis of the top 50 significant metabolites by ANOVA revealed a significant effect of APP/PS1 on plasma metabolism compared to controls. (*C*–*E*) Significant effects of APP/PS1 and OLT1177 treatment were noted with respect to carboxylic acids (*C*), glutaminolysis (*D*), deaminated purines (e.g., allantoate), glutathione turnover metabolites (5-oxoproline), proteolysis (including the urea cycle intermediate ornithine), and tryptophan catabolism (kynurenine) (*E*). (*F*) Polyunsaturated fatty acids were decreased in the bloodstream of APP/PS1 mice and normalized by 7.5 g/kg OLT1177. (*G*) An overview of the overall impact of APP/PS1 and OLT1177 (higher dose) on mouse plasma metabolism is provided in *G*. Data are presented as mean ± SEM. *P < 0.05, **P < 0.01, ***P < 0.001 compared to WT, ^+^*P *< 0.05, ^++^*P *< 0.01, ^+++^*P* < 0.001 compared to APP/PS1 CTRL (one-way ANOVA and with multiple column comparison).

Systems-wide metabolic reprogramming is a hallmark of neurodegenerative diseases and, in particular, AD (23–25). Alteration of circulating levels of carboxylic acids is a recurring trait in pathologies associated with neuroinflammation and microglia activation like AD (26). Increased doses of APP resulting from duplication of chromosome 21 in Down syndrome are associated with an increased incidence of early-onset AD, which is accompanied by plasma metabolic alterations in purine deamination, carboxylic acids, and tryptophan metabolism as a function of inflammatory stimuli (e.g., IFN signaling) (27). To determine whether a similar metabolic reprogramming could be observed in our model, we performed metabolomics analyses of plasma from WT and APP/PS1 mice, either untreated or fed 3.75 or 7.5 g/kg OLT1177 (Fig. 6*A*). Multivariate analyses of metabolomics data, including partial least-square discriminant analysis (Fig. 6*B*) and hierarchical clustering analysis of the top 50 significant metabolites by ANOVA (Fig. 6*C*), revealed a significant effect of APP/PS1 on plasma metabolism compared to controls. Of note, APP/PS1 mice were characterized by increases in several distinctive markers of AD, such as: Carboxylic acids (a circulating marker of mitochondrial dysfunction) (28), deaminated purines (e.g., allantoate), glutaminolysis (glutamine, glutamate), glutathione turnover metabolites (5-oxoproline), proteolysis (including several free amino acids and urea cycle intermediates such as ornithine), and tryptophan catabolism (kynurenine) (Fig. 6 *D*–*G*). Other metabolites like several polyunsaturated fatty acids were decreased in the bloodstream of APP/PS1 mice, further providing correlative evidence between AD and fatty acid metabolism (29). Notably, feeding regimens including OLT1177 at a dose of 3.75 g/kg promoted changes in the levels of these metabolites toward those measured in healthy controls. While the lower dose of OLT1177 was insufficient to normalize the circulating levels for most of these metabolites, the higher dose of 7.5 g/kg significantly improved the metabolic phenotypes and almost completely normalized the levels of the metabolites staed above (Fig. 6*G*).

## Discussion

An appealing option to treat AD or general neurodegenerative diseases is in part based on reducing neuroinflammation. This requires antiinflammatory therapeutic approaches to change the course of the disease (30). Here we report on dapansutrile (OLT1177), a specific NLRP3 inhibitor (18), as a potential oral medication to treat the symptoms of AD.

A suitable model for AD must reflect, in part, the neuropathological and cognitive phenotypes of the disease. The APP/PS1 mouse line, in which Aβ plaques can already be found at the age of 4 to 6 mo and deficits in spatial learning are clearly visible at 8 mo, represents a reliable model (17, 31, 32). To confirm that the APP/PS1 animal model used in this study shows cognitive impairments at the time point of choice (9 mo of age), we performed behavior tests. Indeed, we could corroborate that APP/PS1 mice were impaired in spatial memory performance when compared to WT mice (Fig. 1*F*). This is in line with previous studies with this model, which describe impairment of learning ability in behavioral tests for animals between 8 to 12 mo (33, 34) and 16 to 18 mo (34, 35). However, no deficits were found at the age of 6 mo (34, 35). Therefore, 9-mo-old APP/PS1 mice with impaired learning and memory ability are a suitable model system to approach therapeutic intervention for AD in humans.

Previously, many studies pointed out the important role of neuroinflammation in the progression of AD. Briefly, the initiation of inflammatory signaling pathways favors the release of inflammatory mediators, including cytokines, which in turn can influence neuronal cells and their function (30). Under inflammatory conditions in the brain, glial cells, such as microglia, release cytokines (30, 36). Many of these cytokines (e.g., IL-6, TNF-α, and IL-1β) are linked to the progression of AD (6, 10, 37). It has been shown that higher levels of IL-1β have an effect on tau hyperphosphorylation and thus aggravate AD by impaired LTP and memory formation (38–40). Inhibition of IL-1β signaling, however, contributes to disease-modifying benefits (41). So far, several reports demonstrated increased IL-1β expression in Aβ-plaque–associated microglia cells (40, 42, 43).

Maturation and release of IL-1β in the brain is due to NLRP3 activation (16, 44). Furthermore, it has been reported that the NLRP3 inflammasome might indeed be crucially involved in the immune responses in AD. Halle et al. (14) showed evidence of increased activation of the NLRP3 inflammasome in microglia cells due to Aβ, and in mice with complete NLRP3 inflammasome deletion by genetic manipulation, spatial memory impairment was prevented (16). These findings suggest an important role for the NLRP3 inflammasome in the progression of AD. However, no information is available to date on the effect of NLRP3 inhibition in AD in a therapeutic setting. Therefore, we tested the NLRP3 inhibitor dapansutrile (OLT1177) in APP/PS1 mice using two different oral doses of the inhibitor administered in the animals’ feed. OLT1177 was shown to have beneficial effects in a murine model for acute arthritis by suppressing joint inflammation and in addition reduces the infarct size after ischemia reperfusion injury in the mouse (45–47).

The pharmacokinetics and safety of OLT1177 has been characterized after oral administration to healthy volunteers in a phase 1 trial (17). In addition, oral OLT1177 administered to patients with acute gout flares significantly reduced joint pain as well as plasma cytokines IL-1β and IL-6 (19). In addition, OLT1177 was well tolerated and free of side effects in patients with heart failure; in the cohort receiving 2,000 mg daily, there was an improvement in left ventricular ejection fraction over 14 d (48). Being active orally and well-tolerated, these findings support the potential for OLT1177 to be tested in humans with early cognitive dysfunction.

Inflammation is largely correlated with IL-1β, IL-6, and TNF-α, which tend to induce each other (49). IL-6, which is induced by IL-1, sharply increases under pathological conditions and is significantly increased in the brains of AD patients (37) compared to the healthy adult brain (37, 50). IL-6 may indeed have an important role in AD as it was shown that genetic variation in the IL-6 gene resulted in a delayed onset of the disease (51), and studies suggest that IL-6 plays a role in the synthesis and expression of APP (52). Another cytokine potentially involved in the disease is TNF-α, which was found to be increased in the serum and cortex of AD patients as well as in glial cell cultures after Aβ administration (53–55). A drastic increase in TNF-α levels has been shown to be toxic to cortical neurons (37).

The possible therapeutic effects of OLT1177 for AD was examined using cognitive function assessment in the APP/PS1 mouse and by characterizing underlying cellular mechanisms ranging from systems metabolism to neuronal function, from structural analysis to inflammatory processes. Of note, APP/PS1 mice showed increases in plasma levels of several metabolic markers of AD, such as carboxylic acids, a marker of mitochondrial dysfunction in AD (26). The high dose of OLT1177 (7.5 g/kg) but not the low dose (3.75 g/kg) normalized the circulating levels of these metabolites. Similarly, markers of inflammation-induced purine deamination and proteolysis were increased in APP/PS1 mice—consistent with previous studies in AD (56, 57)—and normalized by OLT1177 at a dose of 7.5 g/kg. Similarly, OLT1177 decreased the levels of oxidant stress markers (e.g., the glutathione turnover marker 5-oxoproline) and kynurenine, a byproduct of tryptophan oxidation. Interestingly, individuals with Trisomy 21, characterized by a higher incidence of early-onset AD, display a similar increase in levels of the metabolites listed above (27) as a result of inflammatory signaling involving the IFN cascade and its downstream target indole 2,3-dioxygenase (IDO1) (27). Since some of the metabolic products of this pathway are neurotoxic (27), it is interesting to note that the circulating levels of these metabolites were normalized by OLT1177. Consistently, the assessments of spatial learning and LTP both exhibit a dose-dependent positive effect of OLT1177. In fact, the lower dose (3.75 g/kg) of OLT1177 had no effect (positive or negative) in APP/PS1 animals. However, when the higher dose is administrated orally, the learning deficits of APP/PS1 animals were rescued.

Microglia cells have been investigated for their potential role in the pathogenesis of neurodegenerative diseases (58, 59). It has been suggested that, in a constant inflammatory environment, more and more microglia may be activated, establishing a situation of chronic neuroinflammation (60). On the one hand, microglia cells are largely protective, clearing the parenchyma of the CNS of cell debris and infectious agents, and are also involved in shaping neuronal connections during postnatal development and supporting structural plasticity during learning processes (61–63). del Río-Hortega (64) has described these cells as phagocytic cells of the CNS, and today it is believed that they are involved in the clearing of Aβ (65). On the other hand, microglia cells react very sensitively to inflammatory processes by changing their morphological shape and have been consequently described as having an “activated” phenotype (36, 65, 66). As such, microglia cells that are attracted to the Aβ plaques show a higher production of proinflammatory cytokines (67, 68). Indeed, in the brain homogenates in our study an increased proinflammatory cytokine production (IL-1β and IL-6) in APP/PS1 animals was demonstrated (Fig. 4 *F* and *G*). Previous studies have shown that CD68 can be described as a microglia activation marker (69, 70), and CD68 has been reported to be elevated in microglial cells associated with Aβ plaques in AD mouse models (71). We also observed an increased activation state using the FACS analysis to measure CD68 levels (Fig. 4 *D* and *E*). We could not find obvious differences in the morphology of nonplaque-associated microglial cells in the hippocampus between WT, untreated APP/PS1 animals, and APP/PS1 animals treated with the NLRP3 inhibitor OLT1177. No analysis of cells could be carried out near plaques. Therefore, the number and activation status of nonplaque-associated microglia was not changed significantly in 9-mo-old APP/PS1 mice.

Finally, to investigate the influence of Aβ-plaque load on the inflammatory phenotype of APP/PS1 mice, we analyzed tissue coimmunostained with IBA-1 (microglial marker) and BAM-10 (Aβ marker). It has been reported that the phagocytic capacity of cortical microglia cells is impaired in APP/PS1 mice (66). Remarkably, we could elucidate a significant reduction in plaques in the area of the cortex APP/PS1 animals fed diet enriched in OLT1177 (7.5 g/kg) compared with APP/PS1 animals fed control food.

Taken together, the data in this study provide strong evidence that the acute, pharmacological inhibition of the NLRP3 inflammasome is well tolerated and can ameliorate neurodegeneration and loss of synaptic plasticity in a murine AD model. The effects of OLT1177 should be further explored in a clinical study in subjects with AD, especially considering that the drug has been demonstrated to be safe in humans at oral doses up to 2,000 mg/d.

## Materials and Methods

For detailed materials and methods used, see *SI Appendix*, *SI Methods*.

### Animals.

Six-month-old male C57BL/6J WT mice and APP/PS1ΔE9 mice were used in this study. Mice were bred and kept under standard housing conditions at the animal facility of Technische Universität Braunschweig, Germany. All experimental procedures had been approved by the responsible authorities (33.19-42502-04-17/2709).

### OLT1177 Treatment.

Six-month-old WT and APP/PS1ΔE9 mice consumed OLT1177 in feed pellets (0, 3.75, or 7.5 g/kg OLT1177 feed) for the treatment duration of 3 mo.

### Cell Culture and LPS Administration.

Postnatal day (P) 3 to P5 mouse brains were removed, homogenized and resuspended in 10 mL culture media (DMEM + 10% FCS + 1% penicillin/streptomycin) in a T-75 flask. The flasks were incubated in a 10% CO_2_ incubator at 37 °C for 2 to 3 wk. The flasks were shaken at 180 rpm for 3 h at 37 °C. Microglia cells were plated in a six-well plate with a density of 10^6^ cells/mL and were treated with 1 µg/mL LPS for 24 h. Thirty minutes after the incubation with LPS, OLT1177 (5 µM or 10 µM) was added. In the last hour of treatment, ATP (5 mM) was added to the cells.

### ELISA.

To determine cytokine levels, mouse IL-1β, IL-6, and TNF-α ELISA kits (R&D Systems) were used according to the manufacturer’s recommendations. Absorbance at 450 nm was measured with an Epoch microplate reader from BioTek and analyzed with the Gen5 software.

### Immunohistochemistry.

Brains were fixed in 4% paraformaldehyde (PFA) for 24 h and then cryoprotected in 30% sucrose solution in PBS (PBS 1×) for 24 h and stored in Tissue-Tek O.C.T. compound (A. Hartenstein Laborversand) at −70 °C. Slices were incubated with anti-ionized calcium-binding adaptor molecule 1 (IBA-1) (1:1,000; rabbit polyclonal, Synaptic System) and clone BAM-10 (1:2,000; monoclonal, Sigma) primary antibodies. The secondary antibodies were Cy3-conjugated AffiniPure Goat Anti-Rabbit IgG (H+L) (1:500; Jackson ImmunoResearch) and Alexa Fluor647-conjugated AffiniPure Goat Anti-Mouse IgG (H+L) (1:500; Jackson ImmunoResearch), which were diluted in PBS 1×.

### FACS Analysis.

A single-cell isolation using the Adult Brain Dissociation Kit (Miltenyi Biotec Order no. 130-107-677) from Miltenyi and the GentleMACS was performed. The cells were resuspended in FACS staining buffer (1× PBS + 1% FCS + 0.1% Na-Azide) and plated in a V-bottom 96-well plate. Cells were stained for 30 min with CD11b-PerCP (1:50), CD45-APC (1:50), and CD68-PE (1:50). The flowcytometry was measured using the BD LRS II SORP and analyzed with FlowJo Software.

### MWM Test.

Spatial memory formation and retention was assessed. Videos were acquired and transmitted to a PC running the tracking software ANY-maze (Stoelting).

### Electrophysiological Experiments.

Electrophysiological recording experiments were performed for different experimental groups. Acute hippocampal slices were prepared. fEPSPs were recorded in the stratum radiatum of the CA1 hippocampal subregion in the acute. For fEPSP recording, the recording electrode (5 MΩ; AM Systems) was positioned in the CA1 apical dendritic layer and signals were amplified by a differential amplifier (Model 1700; AM Systems). Input–output curve (afferent stimulation vs. fEPSP slope) for assessment of basal synaptic transmission and LTP (induced by θ-burst stimulation) was measured. Data acquisition and offline analysis were caried out using IntraCell software (v1.5, LIN) (72, 73).

### Morphological Analysis of Hippocampal Neurons: Golgi-Cox Staining.

For morphological quantification of hippocampal neurons, Golgi-Cox staining (FD rapid Golgi-Cox stain kit) was utilized.

### Imaging and Image Analysis.

Hippocampal neuron morphology within the pyramidal shaped CA1 neurons were imaged (*z*-stack thickness of 0.5 μm) using an Axioplan 2 imaging microscope (Zeiss) equipped with a 63× (N.A. 1) oil objective accompanied with a digital camera (AxioCam MRm, Zeiss).

The microscopic images of anti–IBA-1 and Aβ (clone BAM-10) were taken within the area of cortex and hippocampus. IBA-1 cells were taken from the CA1 area of the hippocampus in three-dimensional (*z*-stack thickness, 1 µm) using Axioplan 2 imaging microscope (Zeiss) equipped with an ApoTome module (Zeiss) with a 20× objective (NA, 0.8) and a digital camera (AxioCam MRm; Zeiss). To analyze, a region of interest was drawn in ImageJ software (Wayne Rasband, NIH, Bethesda, MD).

### Metabolomics Analyses.

Plasma (10 µL) metabolomes were characterized by ultrahigh-pressure liquid chromatography coupled to high-resolution mass spectrometry (Vanquish–Q Exactive, Thermo Fisher). Extraction was performed at a 1:10 ratio in ice-cold methanol:acetonitrile:water 8:3:2 (vol/vol) via vortexing for 30 min at 4 °C.

### Statistical Analysis.

Data were analyzed and plotted by GraphPad Prism 6 (GraphPad Software) and presented as mean ± SEM. Differences in dendritic spine density, immunostaining, and cytokines measurement data were subjected to a one-way ANOVA, whereas two-way ANOVA was used for behavioral and electrophysiological experiments. Fisher’s LSD, Bonferroni’s, and Tukey’s multiple comparisons were used as a post hoc test, depending on experiments. The minimum significance value was considered as *P* < 0.05. All statistical analysis and number of different experimental groups are reported in the figure legends.

## Supplementary Material

Supplementary File

## Data Availability

All study data are included in the article and supporting information.
