# Supplementary material for: The NLRP3 inflammasome inhibitor OLT1177 rescues cognitive impairment in a mouse model of Alzheimer’s disease
Source: Proc Natl Acad Sci U S A. 2020 Nov 30;117(50):32145–54. doi: 10.1073/pnas.2009680117 (PMC7749353; doi:10.1073/pnas.2009680117)
Supplement: Supplementary File [file pnas.2009680117.sapp.pdf]

PNAS
www.pnas.org

Supplementary Information for

The NLRP3 inflammasome inhibitor OLT1177 rescues cognitive impairment in a mouse model of Alzheimer's disease

Niklas Lonnemann[a], Shirin Hosseini[a,b], Carlo Marchetti[c], Damaris B. Skouras[d], Davide Stefanoni[e], Angelo D'Alessandro[e], Charles A. Dinarello[c,f#] and Martin Korte[a,b#]

[a]Department of Cellular Neurobiology, Zoological Institute, TU Braunschweig, 38106 Braunschweig, Germany

[b]Helmholtz Centre for Infection Research, Neuroinflammation and Neurodegeneration Group, 38124 Braunschweig, Germany

[c]Department of Medicine, University of Colorado Denver, Aurora, CO 80045, USA

[d]Olatec Therapeutics LLC, New York, New York 10065, USA

[e]Department of Biochemistry and Molecular Genetics, University of Colorado Denver, Aurora, CO 80045, USA

[f]Department of Medicine, Radboud University; Medical Center, 6525 Nijmegen, The Netherlands

#Corresponding Authors :

Martin Korte, PhD
E-Mail : m.korte@tu-bs.de
Zoological Institute
Div. Cellular Neurobiology
TU Braunschweig
Spielmannstr. 7
38106 Braunschweig
Deutschland

Charles A. Dinarello, MD
E-Mail : cdinare333@aol.com
Department of Medicine
University of Colorado Denver
Aurora
CO 80045
USA

ORCID : Martin Korte ORCID ID 0000-0001-6956-5913

ORCID : Charles A. Dinarello ORCID ID 0000-0002-5073-8316

www.pnas.org/cgi/doi/10.1073/pnas.2009680117

This PDF file includes:

Supplementary text

Figures S1

SI References

Supplementary Information Text

SI Appendix Methods

Animals. 6 months old male C57BL/6J wild-type mice (WT) and APP/PS1ΔE9 mice were used in this study. Mice were bred and kept under standard housing conditions at the animal facility of TU Braunschweig, Germany. All experimental procedures had been approved by the responsible authorities (33.19-42502-04-17/2709).

OLT1177 treatment. 6 months old WT and APP/PS1ΔE9 mice consumed OLT1177 in feed pellets (0, 3.75 or 7.5 mg/g) for the treatment duration of 3 months. Characterization for treatments as followed, WT mice treated with control food (referred as WT), WT treated with 3.75 g/kg OLT1177 (referred as WT 3.75 g/kg), WT treated with 7.5 g/kg OLT1177 (referred as WT 7.5 g/kg), APP/PS1 mice treated with control food (referred as APP/PS1), APP/PS1 treated with 3.75 g/kg OLT1177 (referred as APP/PS1 3.75 g/kg), APP/PS1 treated with 7.5 g/kg OLT1177 (referred as APP/PS1 7.5 g/kg).

Cell culture and LPS administration. For culture preparation. Neonatal mouse brains aged between P3-P5 were used. Briefly, the meninges were removed and the brain transferred into HBSS 1X on ice. With a 10 ml pipette the tissue was transferred into a sterile 50 ml conical tube and centrifuged at 2000 rcf for 5 min at 4°C. The pellet was re-suspended in 5 ml fresh HBSS 1X and applied on a cell strainer (100 µm pores). After centrifugation at 2000 rcf for 5 min at 4°C the pellet was re-suspended in 10ml culture media (DMEM + 10% FCS + 1% Penicillin/Streptomycin) and transferred into a T-75 flask. The flasks were incubated in a 10% CO_2 incubator at 37°C for 2-3 weeks. Cell culture was first incubated for 3 days, then media was replaced 50% of fresh culture media. In the following every 7 days the media was replaced. After mixed culture was completely confluent, the flasks were shaken at 180 rpm for 3 h at 37°C. The media was collected (without disrupting the astrocyte layer on the flask surface) and centrifuged at 3000 rpm for 10 min at room temperature (RT). Microglia cells were plated in 6-well plate with a density of 10^6 cells/ml and were treated with 1 µg/ml LPS for 24h. 30 min after the incubation with LPS, OLT1177 (5 µM or 10 µM) was added. In the last hour of treatment, ATP (5 mM) was added to the cells.

ELISA. To quantify the presence of proinflammatory cytokines, including IL-1β, IL-6 and TNF-α in the supernatant of homogenized brain hemispheres of mice, an ELISA experiment was performed. For this purpose, mice were deeply anesthetized with CO_2 and killed via decapitation. For protein isolation, the tissue was homogenized in 400 µl of STKM lysis buffer containing the protease inhibitor mixture (cOmplete). The samples were centrifuged at 4°C for 10 min at 13000 × g. The supernatant was collected and stored at −70°C. To determine cytokine level, mouse IL-1β, IL-6 and TNF-α ELISA kits (R&D Systems) were used according to the manufacturer's recommendations. Absorbance at 450 nm was measured with an Epoch microplate reader from BioTek and analyzed with the Gen5™ software.

Immunohistochemistry. To determine the morphological phenotype of microglia cells and to analyze the amount of amyloid-beta plaques, the brains were isolated and fixed in 4% paraformaldehyde (PFA) for 24 hours and then cryoprotected in 30% sucrose solution in phosphate buffered saline (PBS 1X) for 24 hours and stored in Tissue-Tek® O.C.T.™ compound (A. Hartenstein Laborversand) at -70°C. For fluorescence immunostaining, 30 µm brain slices were cut using the Cryostat. Sections were washed in 1x PBS and blocked in 1x PBS solution containing 0.2% Triton X-100, 5% goat serum, 5% donkey serum and 1% BSA for 1 hour at room temperature. Using the free floating method, slices were incubated overnight at 4°C with anti-ionized calcium-binding adaptor molecule 1 (IBA-1) (1:1000; rabbit polyclonal,

Synaptic System), clone BAM-10 (1:2000; monoclonal, SIGMA) primary antibodies diluted in PBS 1X, 0.2% Triton X-100 and 5% goat serum / 5% donkey serum. The secondary antibodies were Cy™3-conjugated AffiniPure Goat Anti-Rabbit IgG (H+L) (1:500; Jackson ImmunoResearch) and Alexa Fluor®647-conjugated AffiniPure Goat Anti-Mouse IgG (H+L) (1:500; Jackson ImmunoResearch) which were diluted in PBS 1X. Sections were washed with PBS 1X and stained with 4',6-diamidino-2-phenylindole (DAPI) (SIGMA) followed by cover-slipping with Fluoro-gel with Tris buffer (Electron Microscopy Sciences).

FACS analysis. FACS was used to analyze the microglia activation marker CD68. The brains were collected from the OLT1177 treated animals. A single cell isolation using the Adult Brain Dissociation Kit (Miltenyi Biotec Order no. 130-107-677) from Miltenyi and the GentleMACS was performed. The cells were resuspended in FACS staining buffer (1xPBS + 1% FCS + 0.1% Na-Azide) and plated in V-bottom 96-well plate. Cells were stained for 30min with CD11b-PerCP (1:50), CD45-APC (1:50), CD68-PE (1:50). The flowcytometry was measured using the BD LRS II SORP and analyzed with FlowJo Software.

Morris Water Maze test. Spatial memory formation and retention was assessed using the Morris water maze (MWM) assay (1). In this test, a 10 cm escape platform was submerged 1 cm below the water surface into a circular plastic pool (160 cm in diameter and 60 deep) filled up to 30 cm with opaque water (titanium dioxide, Euro OTC Pharma; water temperature 19-20 °C). Three visual cues were positioned on the walls around the pool. All tests were performed under dim light at the same time of day during the light period between 9:00 to 16:00 by an experimenter blind to all groups. A digital camera was installed above the center of the maze and videos were acquired and transmitted to a PC running the tracking software ANY-maze (Stoelting, USA). Prior to the training, a visible platform task was performed as a pre-training and used to ensure that swimming ability and visual acuity were intact in all experimental groups, moreover, this phase was important for the animals to get accustomed to the test situation. During this phase, the animals had two trials (maximum of 60 s each) per day for three consecutive days to reach the visible platform, the position was alternated during the trials as is standard practice.

Subsequently, training in the Morris water maze was performed for 8 days with the invisible platform located in the northwest (NW) quadrant. Each day, animals were placed in the water for four trials, with different starting points (SW, S, E, and NE) randomly at a 5 min intertrial interval. The animals were permitted to swim freely for 60 s or until the platform was reached. Otherwise, they were guided to the platform and allowed to sit on it for 15 s. For evaluation of reference memory, two probe trial tests were performed at the third day of the acquisition training, prior to the training session on that day. Another reference memory test was performed 24 h after the last day of acquisition training (day 9). During the probe trial, the platform was removed and the animals were allowed to swim freely for 45 s (starting position SE).

Electrophysiological experiments. To study the function of CA1 hippocampal neurons, electrophysiological recording experiments were caried out in different experimental groups. For this purpose, acute hippocampal slices were prepared from mice in four groups including wild-type, APP/PS1, APP/PS1 treated with 3.75 g and APP/PS1 treated with 7.5 g of OLT1177. Briefly, mice were deeply anesthetized with 100% CO_2, killed, and then brains were quickly removed and transferred into ice-cold carbogenated (95% O_2 and 5% CO_2) artificial CSF (ACSF) containing the following (in mM): 124 NaCl, 4.9 KCl, 1.2 KH_2PO_4, 2.0 $MgSO_4$, 2.0 $CaCl_2$, 24.6 $NaHCO_3$, and 10 D-glucose, pH 7.4. Afterward, the hippocampus was dissected and transverse hippocampal slices (400 μm) were obtained using a manual tissue chopper. The whole procedure was done in 2–3 min. The hippocampal slices were transported to an interface recording chamber (Scientific System Design) and they were incubated at 32°C with a constant flow rate (0.5 ml/min) of carbogenated ACSF for 2 h prior to the start of recordings. Field excitatory post synaptic potentials (fEPSPs) were recorded in the stratum radiatum of the hippocampal CA1 sub-region. Responses were evoked by stimulation of the Schaffer collateral pathway using two monopolar, lacquer-coated stainless-steel electrodes (5 MΩ; AM Systems). These stimulation electrodes (S1 and S2) were placed equidistantly on both sides of the recording electrode and, by this means, two independent stimulation pathways could be used for the same CA1 recording region. For recording fEPSPs (measured as the first slope function), the recording electrode (5 MΩ; AM Systems) was positioned in the CA1 apical dendritic layer (at least 20 μm away from the stratum pyramidale) and signals were amplified by a differential amplifier (Model 1700; AM Systems). The

signals were digitized using a CED 1401 analog-to-digital converter (Cambridge Electronic Design). An input–output curve (afferent stimulation vs fEPSP slope) for assessment of basal synaptic transmission was generated after the pre-incubation period. Test stimulation intensity was modified to be adjusted to extract fEPSP slope as 40% of the maximal fEPSP response for both synaptic inputs S1 and S2. To investigate long-term potentiation, 20 min after baseline recording, LTP was induced by theta-burst stimulation (TBS) including four bursts at 100 Hz repeated 10 times in a 200 ms interval. This stimulation was repeated three times in a 10 s interval. Only healthy sections with a stable baseline were included in the electrophysiological data analysis. The slope of fEPSPs was measured over time for 60 min and normalized to the baseline. Data acquisition and offline analysis were caried out using IntraCell software (version 1.5, LIN) (2, 3).

Morphological analysis of hippocampal neurons: Golgi-Cox staining. For morphological quantification of hippocampal neurons, Golgi-Cox staining was performed. For this purpose, mice were deeply anesthetized with CO_2 and sacrificed. The left hemisphere was incubated in FD rapid Golgi-Cox stain kit according to the manufacturer's protocol. Briefly, each hemisphere was immersed in 2 ml impregnation Golgi solution, a mixture of solutions A (potassium dichromate and mercuric chloride) and B (potassium chromate) for at least 14 days to 1 month at room temperature in the dark followed by one week in tissue-protectant solution C at room temperature. Both impregnation and solution C were replaced with fresh solutions on the second day. Subsequently, hemispheres were blocked in 2% agar and using a vibratome (Leica VT 1000 S) 200 µm thick coronal sections were cut. The slices were collected on gelatin-coated glass slides. Afterward, the sections were processed with solutions D and E for signal development for 10 minutes before being dehydrated through graded alcohols and mounted using Permount (Thermo Fisher Scientific). The slides were then kept in a horizontal position for drying in the dark for at least 48 h before imaging (2, 3).

Imaging and image analysis. To survey the hippocampal neuron morphology, second- or third-order branches of both apical and basal dendrites within the pyramidal shaped CA1 neurons (8 cells in each subregion per animal) were imaged in three-dimensions (z-stack thickness of 0.5 µm) using an Axioplan 2 imaging microscope (Zeiss) equipped with a 63x (N.A. 1) oil objective accompanied with a digital camera (AxioCam MRm, Zeiss). In all selected neurons, spine density per micrometer of dendrites was calculated using Fiji software (BioVoxxel) on the segments of dendritic branches with a length of more than 60-70 µm which were positioned at least 40-50 µm away from the cell soma. The total number of spines along the segment of dendritic branches was counted manually. All slides were coded and analyses were performed blindly (2, 3).

The microscopic images of anti-IBA-1 and amyloid-β (clone BAM-10) were taken within the area of cortex and hippocampus (3 slices per animal). To survey the hippocampal microglial morphology, images of IBA-1 cells were taken from the CA1 area of the hippocampus in 3D (z-stack thickness 1 µm) using Axioplan 2 imaging microscope (Zeiss) equipped with an ApoTome module (Zeiss) with a 20X objective (NA, 0.8) and a digital camera (AxioCam MRm; Zeiss). To analyze the density of microglia cells, a region of interest (ROI) was drawn in each frame of the hippocampus and the IBA-1 positive cells were determined with the clearly visible nuclei by DAPI and ImageJ software (Wayne Rasband NIH, USA). To investigate the activation status of microglia cells primary processes of IBA-1 positive cells were counted with ImageJ software.

Metabolomics analyses. Plasma (10 µl) metabolomes were characterized by ultra-high pressure liquid chromatography coupled to high resolution mass spectrometry (Vanquish – Q Exactive, Thermo Fisher, San Jose, CA). Extraction was performed at a 1:10 ratio in ice cold methanol:acetonitrile:water 8:3:2 *v/v* via vortexing for 30 min at 4°C, as previously described (4). Analytical details of chromatographic gradients and instrument operation settings were identical to those described extensively in prior methodological and application papers (4, 5) and are here omitted in the interest of space.

Statistical analysis. Data were analyzed and plotted by GraphPad Prism 6 (GraphPad Software, Inc. USA) and presented as mean±SEM. Differences in dendritic spine density, immunostaining and cytokines measurement data were subjected to a one-way ANOVA whereas two-way ANOVA was used for behavioral and electrophysiological experiments. Fisher's LSD, Bonferroni's and Tukey's multiple comparisons were used as a post hoc test depending on experiments. The minimum significance value was considered as $p < 0.05$. All statistical analysis and n of different experimental groups were reported in the results and figure legends respectively.

SI Appendix Extended Figure legends

Figure 1: Oral administration of OLT1177 in APP/PS1 animals for 3 months restores cognitive deficits. (A) The treatment of either OLT1177 (3.75 g/kg and 7.5 g/kg; drug per kg feed) or control food was started at the age of 6 months and continued for 3 months. (B and C) During the treatment period neither WT nor APP/PS1 mice show differences in weight (N = number of animals WT N = 16, WT low N = 17, WT high N = 19; APP/PS1 N = number of animals APP/PS1 N = 30, APP/PS1 low N = 27, APP/PS1 high N = 33). (D) WT and APP/PS1 mice indicate a learning behavior during the training phase of the spatial learning test. APP/PS1 mice show higher escape latency during acquisition on day 5 compared to WT mice (WT: One-way ANOVA : $F (7, 48) = 4.268$, $p = 0.001$; APP/PS1: One-way ANOVA : $F (7, 72) = 2.96$, $p = 0.008$; APP/PS1 3.75 g/kg: One-way ANOVA : $F (7, 72) = 5.35$, $p < 0.001$; APP/PS1 7.5 g/kg: One-way ANOVA : $F (7, 88) = 6.875$, $p < 0.001$ Fig. 1D; Two-way RM ANOVA : $F (3, 35) = 3.975$, $p = 0.015$, D: Bonferroni's multiple comparison: Day 5 $p = 0.037$; N = number of animals WT N = 7, APP/PS1 CTRL N = 10, APP/PS1 low N = 10, APP/PS1 high N = 12). (E) Moreover, on day 3 and 5 the APP/PS1 mice show increased escape latency compared to APP/PS1 mice treated with 7.5 g/kg OLT1177 (One-way ANOVA : FDay3 (3, 35) = 7.41, $p = 0.001$; FDay5 (3, 35) = 4.63, $p = 0.007$; Bonferroni's multiple comparison: Day 3: $p = 0.001$; day 5 $p = 0.007$, E: N = number of animals WT N = 7, APP/PS1 CTRL N = 10, APP/PS1 low N = 10, APP/PS1 high N = 12). (F) The reference test was performed 24 h after the last training session. WT mice and APP/PS1 mice treated with 7.5 g/kg OLT1177 display a significant preference for the Target Quadrant (TQ), whereas the APP/PS1 mice with control or low dose food did not show any preference (One-way ANOVA : $F (7, 70) = 11.57$, $p < 0.001$; Turkey's multiple comparison NT vs TQ: WT $p < 0.001$; APP/PS1 $p > 0.99$; APP/PS1 3.75 g/kg $p = 0.39$; APP/PS1 7.5 g/kg $p < 0.001$, Fig. 1F; One-way ANOVA : $F (7, 70) = 11.57$, $p < 0.001$; Turkey's multiple comparison; WT vs APP/PS1 $p = 0.001$; WT vs APP/PS1 3.75 g/kg $p = 0.014$; WT vs APP/PS1 7.5 g/kg $p = 0.68$; One-way ANOVA : $F (7, 70) = 11.57$, $p < 0.001$; Turkey's multiple comparison; APP/PS1 vs APP/PS1 7.5 g/kg $p = 0.008$, N = number of animals WT N = 7, APP/PS1 CTRL N = 10, APP/PS1 low N = 10, APP/PS1 high N = 12). (G) WT animals treated with control food, low dose or high dose OLT1177 did not show any differences between the groups. All WT mice demonstrate a significant preference for the TQ (N = number of animals WT CTRL N = 7, WT low N = 8, WT high N = 8). (H) The heat maps of pooled animals manifest the results of the reference test (scale blue 0 s to red 1.9 s, N = number of animals WT N = 4, APP/PS1 CTRL N = 3, APP/PS1 low N = 3, APP/PS1 high N = 3). Data are presented as mean ± SEM. *** $p < 0.001$ compared to WT, +++ $p < 0.001$ compared to APP/PS1 CTRL, ^^^ $p < 0.001$ compared to NT.

Figure 2: Oral administration of OLT1177 in APP/PS1 animals for 3 months rescues synaptic plasticity impairment to WT conditions. (A and B) WT and APP/PS1 mice treated either with control food or OLT1177 did not show any differences in the properties of basal synaptic transmission (WT: Two-way RM ANOVA : $F (2, 82) = 0.32$, $p = 0.72$, Fig. 2A; Two-way RM ANOVA : $F (3, 118) = 0.275$, $p = 0.843$, B) and (C and D) ratios of pared-pulse facilitation (WT: Two-way RM ANOVA : $F (2, 80) = 1.51$, $p = 0.22$, Fig. 2C; Two-way RM ANOVA : $F (3, 105) = 1.61$, $p = 0.19$, D). (E) WT mice treated either with control food or OLT1177 exhibited the same magnitude of LTP in response to strong afferent stimulation (LTP was induced by theta-burst stimulation: TBS, four bursts at 100 Hz repeated 10 times in a 200 ms interval, repeated three times in a 10 seconds interval; denoted with an arrow) (Two-way RM ANOVA: $F (2, 73) = 0.66$, $p = 0.51$). (F) Comparison of mean values (average of last 5 minutes of recordings) LTP magnitude in control mice summarized as bar graphs (One-way ANOVA : $F (2, 73) = 0.64$, $p = 0.52$). (G) APP/PS1 mice treated with either control food or low dose of OLT1177 showed a significant impairment in LTP induced by theta-burst stimulation (TBS) compared to control group, however, administration of OLT1177 at high dose could rescue the phenotype in APP/PS1 mice (Two-way RM ANOVA : $F (3, 116) = 10.89$, $p < 0.001$). (H) The mean LTP magnitude (average of 55-60min after TBS) was significantly lower in APP/PS1 mice treated either with control food ($p < 0.001$) or 3.75 mg/kg of OLT1177 ($p < 0.001$) compared to WT. Administration of OLT1177 at high dose could rescue the phenotype in APP/PS1 mice (CTRL: 1.661 ± 0.03, APP/PS1: 1.27 ± 0.05, APP/PS1-low dose: 1.23 ± 0.05 and APP/PS1-high dose: 1.52 ± 0.06, One-way ANOVA : $F (3, 116) = 11.44$, $p < 0.001$, Turkey's multiple comparison: WT vs APP/PS1 $p = 0.001$, WT vs APP/PS1 3.75 g/kg $p = 0.001$, WT vs APP/PS1 7.5 g/kg $p = 0.41$, APP/PS1 vs APP/PS1 7.5 g/kg $p = 0.007$). Data are presented as mean ± SEM. *** p

< 0.001 compared to WT, ++ p < 0.001 compared to APP/PS1 (Number of mice in each group = 6 - 7 and number of slices in each group = 24 - 36).

Figure 3: Oral administration of OLT1177 in APP/PS1 animals for 3 months restores dendritic spine loss to WT conditions. (A and B) Dendritic spine density of CA1- apical and basal was not changed in WT mice following administration of 3.75 g/kg and 7.5 g/kg OLT1177 (One-way ANOVA : F (2, 71) = 0.32, p = 0.72, A; One-way ANOVA : F (2, 71) = 1.79, p = 0.17, B). (C and D) Spine density in both apical and basal dendrites of CA1 hippocampal neuron was significantly diminished in APP/PS1 mice treated either with control food (p < 0.001) or 3.75 g/kg OLT1177 (p < 0.001) compared to WT. Administration of 7.5 g/kg OLT1177 could rescue the phenotype in APP/PS1 mice (One-way ANOVA : F (3, 140) = 27.65, p < 0.001, Turkey's multiple comparison: WT vs APP/PS1 p = 0.001, WT vs APP/PS1 3.75 g/kg p = 0.001, WT vs APP/PS1 7.5 g/kg p = 0.89, APP/PS1 vs APP/PS1 7.5 g/kg p = 0.001 C; (One-way ANOVA : F (3, 140) = 20.98, p < 0.001, Turkey's multiple comparison: WT vs APP/PS1 p = 0.001, WT vs APP/PS1 3.75 g/kg p = 0.001, WT vs APP/PS1 7.5 g/kg p = 0.36, APP/PS1 vs APP/PS1 7.5 g/kg p = 0.001 D). Representative images of dendritic spines of hippocampal CA1 neurons in the tested groups are presented (Scale bar = 5 μm). Data are presented as mean ± SEM. *** p < 0.001 compared to WT, +++ p < 0.001 compared to APP/PS1 (Number of mice in each group = 5 and number of dendrites in each group = 8).

Figure 4: The administration of oral OLT1177 in APP/PS1 animals for 3 months significantly reduces microglia activation. (A-C) Evaluation of microglial cells by immunostaining of IBA-1 (A; IBA-1 in red, DAPI in blue; scale bar 100 μm) showed no significant differences in activation status of microglial cells between APP/PS1 animals treated with OLT1177 or without (n = number of sample APP/PS n = 9, APP/PS1 low n = 9, APP/PS1 high n = 6, B; n = number of sample APP/PS1 n = 18, APP/PS1 low n = 18, APP/PS1 high n = 12, C). (D and E) In addition the percentage of CD68 expressing cells, as an activation marker for microglial cells, was enhanced in CD11b+/CD45low gated cells of APP/PS1 mice treated with control food (One-way ANOVA : F(3, 13) = 2.86; p = 0.077; Fisher's LSD multiple comparison: WT vs APP/PS1 p = 0.015, N = number of animals WT N = 5, APP/PS1 CTRL N = 4, APP/PS1 3.75 g/kg N = 4, APP/PS1 7.5 g/kg N = 4). (F-H) Elevation of pro-inflammatory cytokines IL-1β, IL-6 and TNF-α was detected in brain homogenates of APP/PS1 mice (IL-1β One-way ANOVA : F(3, 34) = 8.15, p = 0.001; Bonferroni's multiple comparison; WT vs APP/PS1 p = 0.019; APP/PS1 vs APP/PS1 7.5 g/kg p = 0.001, F; IL-6 One-way ANOVA : F(3, 34) = 8.52, p = 0.001; Bonferroni's multiple comparison; WT vs APP/PS1 p = 0.039; APP/PS1 vs APP/PS1 7.5 g/kg p = 0.01, G; TNF-α One-way ANOVA : F (3, 16) = 3.34, p = 0.045; Bonferroni's multiple comparison: APP/PS1 vs APP/PS1 7.5 g/kg OLT1177 p = 0.011 H; n = number of sample WT n = 6, APP/PS1 CTRL n = 10, APP/PS1 3.75 g/kg n = 12, APP/PS1 7.5 g/kg n = 10). (I) The amount of Aβ plaques was determined using immunostaining for IBA-1 and BAM-10 (clone for amyloid-β) (IBA-1 in green, DAPI in blue, BAM-10 in red; scale bar 500 μm). (J) Plaque load was lower in the cortex of the APP/PS1 animals fed with 7.5 g/kg OLT1177 compared to APP/PS1 animals (Cortex Unpaired t-test: t = 2.476, df = 10, p = 0.03; Hippocampus: Unpaired t-test: t = 1.484, df = 10, p = 0.16, n = number of sample n = 6 in both groups). Data are presented as mean ± SEM. *** p < 0.001 compared to WT, +++ p < 0.001 compared to APP/PS1 CTRL.

Figure 5: Direct effects of OLT1177 on microglia cells in vitro. WT primary microglia cells were cultured and stimulated with 1 μg/ml LPS (E.coli) with or without OLT1177 (5 μM or 10 μM) for 24 hours. (A-C) The pro-inflammatory cytokines IL-1β, IL-6 and TNF-α were measured in the supernatant. Microglia cells treated with 5 μM OLT1177 showed a significantly reduced release of all three cytokines compared to cells treated with LPS alone (IL-1β: One-way ANOVA : F(3, 41) = 29.73, p < 0.001; Turkey's multiple comparison LPS vs LPS 5 μM OLT p = 0.02; LPS vs LPS 10 μM OLT p = 0.01, A; IL-6: One-way ANOVA : F(3, 47) = 173.9, p < 0.001; Turkey's multiple comparison LPS vs LPS 5 μM OLT p = 0.001, B; LPS vs LPS 10 μM OLT p = 0.57, Fig. 5B; TNF-α: One-way ANOVA : F(3, 56) = 75.78, p < 0.001; LPS vs LPS 5 μM OLT p = 0.006; LPS vs LPS 10 μM OLT p = 0.054, C). Data are presented as mean ± SEM. *** p < 0.001. N = 3 independent rounds, n = 7-20 samples.

Figure 6: APP/PS1 mice show increases in plasma metabolic markers of AD that are normalized by OLT1177 treatment. Metabolomics analyses were performed on plasma from WT and APP/PS1 mice, either untreated or fed 3.75 or 7.5 g/kg OLT1177. (A) Multivariate analyses of metabolomics data, including partial least square-discriminant analysis and (B) hierarchical clustering analysis of the top 50

significant metabolites by ANOVA revealed a significant effect of APP/PS1 on plasma metabolism compared to controls. (C-E) Significant effects of APP/PS1 and OLT1177 treatment were noted with respect to carboxylic acids (C), glutaminolysis (D), deaminated purines (e.g., allantoate), glutathione turnover metabolites (5-oxoproline), proteolysis (including the urea cycle intermediate ornithine), and tryptophan catabolism (kynurenine – E). (F) Polyunsaturated fatty acids were decreased in the bloodstream of APP/PS1 mice and normalized by 7.5 g/kg OLT1177. (G) An overview of the overall impact of APP/PS1 and OLT1177 (higher dose) on mouse plasma metabolism is provided in H. Data are presented as mean ± SEM. *** $p < 0.001$ compared to WT, +++ $p < 0.001$ (one-way ANOVA and with multiple column comparison) N = 3 animals.

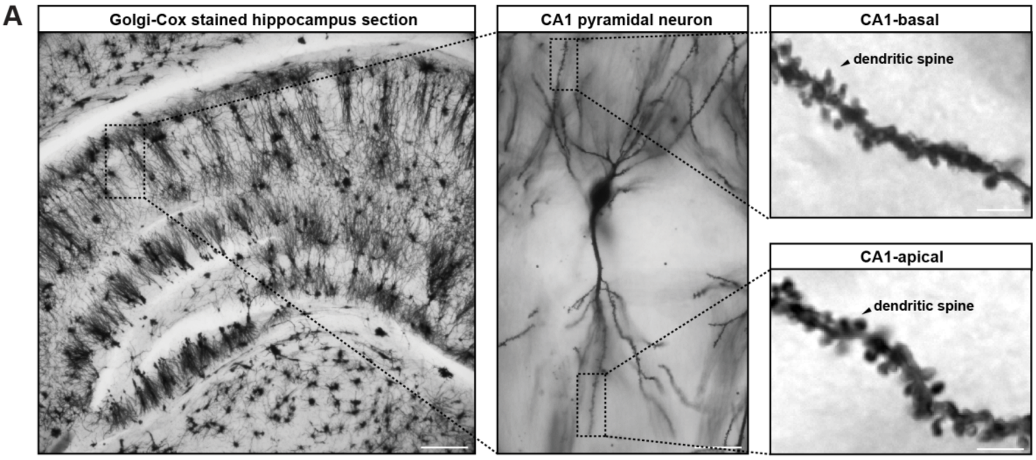

Figure S1: (A) Representative images of Golgi-stained hippocampus section (Scale bar = 100 µm), CA1 neuron (Scale bar = 20 µm) and spines in CA1 apical and basal dendrites (Scale bar = 5 µm).

SI Referencfes

1. R. Morris, Developments of a water-maze procedure for studying spatial learning in the rat. *J. Neurosci. Methods* **11**, 47-60 (1984).
2. S. Hosseini *et al.*, Long-term neuroinflammation induced by influenza A virus infection and the impact on hippocampal neuron morphology and function. *J. Neurosci.* **38**, 3060-3080 (2018).
3. S. Hosseini *et al.*, Type I Interferon Receptor Signaling in Astrocytes Regulates Hippocampal Synaptic Plasticity and Cognitive Function of the Healthy CNS. *Cell Rep.* **31**, 107666 (2020).
4. J. A. Reisz, C. Zheng, A. D'Alessandro, T. Nemkov, "Untargeted and semi-targeted lipid analysis of biological samples using mass spectrometry-based metabolomics" in High-throughput metabolomics. (Springer, 2019), pp. 121-135.
5. G. Cavalli *et al.*, Interleukin 37 reverses the metabolic cost of inflammation, increases oxidative respiration, and improves exercise tolerance. *Proc. Natl. Acad. Sci. U.S.A.* **114**, 2313-2318 (2017).
